# Huge omental lymphangioma with haemorrhage in children: case report

**DOI:** 10.11604/pamj.2020.35.20.8585

**Published:** 2020-01-24

**Authors:** Abdelhalim Mahmoudi, Mohammed Rami, Khalid Khattala, Aziz El Madi, Youssef Bouabdallah

**Affiliations:** 1 Department of Pediatric Surgery, CHU Hassan II, Faculty of Medicine and Pharmacy, Sidi Mohamed Ben Abdellah University, Fez, Morocco

**Keywords:** Abdominal, omental lymphangioma, childhood, surgery

## Abstract

Omental cystic lymphangioma is a rare benign intraabdominal anomaly with uncertain etiology, predominantly occurring in children. Most cases of abdominal lymphangioma are asymptomatic. However, patients may occasionally present with acute abdomen because of an intestinal obstruction or peritonitis caused by infected cysts, hemorrhaging, and/or torsion. This report describes a case of omental cystic lymphangioma with acute intracystic haemorrhage. Ultrasonography and computed tomography (CT) scan confirmed the diagnosis. Complete excision of the cyst along without omentectomy done with no clinical or radiological evidence of recurrence till 17 months.

## Introduction

A lymphangioma is a benign proliferation of lymph vessels, producing fluid-filled cysts that result from a blockage of the lymphatic system. Lamphangioma is located preferentially in head, neck, and axilla in children. However, lymphangiomas in abdomen are extremely rare. They account for 3% [1] to 9.2% [2] of all pediatric lymphangiomas. From 50% to 60% of cystic lymphangioma cases present symptoms by age 1 year and 90% of the cases present by age 2 years [3]. Abdominal cystic lymphangiomas arise from the mesentery (59% - 68%), omentum (20% - 27%), and retroperitoneum (12% - 14%) [4, 5] and most clinical presentations are due to mesenteric lymphangiomas. Complicated cases have been reported. Cases of abdominal lymphangioma present acute abdomen; attributed to intestinal obstruction [1] with volvulus [6], extrinsic compression and entrapment or peritonitis caused by rupture [7], torsion, haemorrhage into the cyst [8] or an infected cyst. Complete resection is the treatment of choice and has an excellent prognosis. Although an abdominal lymphangioma is considered benign, it may become locally invasive. Therefore, any involved organ must also be resected. Incomplete resection may lead to recurrence. Follow-up imaging is advised, with ultrasound as the modality of choice.

## Patient and observation

A 3-year-old boy presented with huge abdominal swelling, he had experienced 2 months of progressive abdominal distension and had to walk bent over for a few days prior to admission. He had experienced abdominal pain or vomiting and although eating required a long time, it was nevertheless still possible. On physical examination, tachycardia, abdominal distension, and scarce bowel sounds were found. No mass could be palpated. The laboratory data presented anemia (6g/dl).

**Figure 1:**
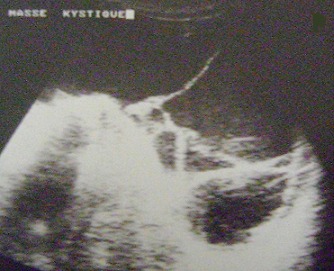
Ultrasonographic examination demonstrated a giant abdominal cystic lesion with internal echogenic particles and septations

**Figure 2:**
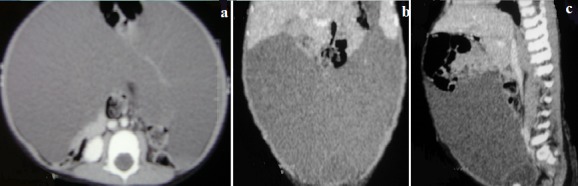
Computed tomography images shows a huge abdominal cystic mass measuring 35cm x 25cm x 15, with a septum that occupied nearly the entire abdominal cavity: A) transversal section; B) coronal section; C) sagittal section

The abdominal x-ray was unremarkable. The ultrasonographic examination demonstrated a giant cystic lesion with internal echogenic particles and septations that extended from epigastrium to pelvis, displacing all retroperitoneal and intraperitoneal organs posteriorly with clear margins between visceral organs (Figure 1). No apparent sonographic abnormality was detected in the internal organs, except compression due to the cyst. A CT examination disclosed the huge cystic lesion with homogenous density that entirely filled the abdominal cavity measuring 35 cm×25 cm×15 cm (Figure 2 A, Figure 2 B, Figure 2 C). With all these clinical and imaging findings, the provisional diagnosis of omental or mesenteric cystic lymphangioma was considered.

**Figure 3:**
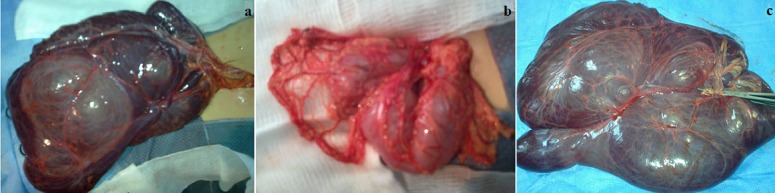
A) Intraoperative findings revealed a cystic mass, 30cm in diameter, in continuity with the greater omentum, the cyst contained serosanguinous fluid; B) the lesion was completely excised without any other combined resection; C) resected specimen shows the huge cystic lymphangioma

At surgery, a huge, soft cystic mass lesion with intracystic haemorrhage and smooth surface was found in the greater omentum, totally filling the abdominal cavity and displacing all intraabdominal viscera posteriorly, that was not adherent to the neighboring structures. The cystic lesion was resected totally; no bowel or other organ resection was done (Figure 3 A, Figure 3 B, Figure 3 C). The patient was discharged 3 days after the operation with an uneventful recovery. Anatomopathologic examination demonstrated a dilatation of the lymphatic vessels without evidence of neoplastic cells, compatible with a cystic lymphangioma.

## Discussion

Lymphangioma is a benign lesion, with more than 80% of cases occurring during childhood [9, 10]. It usually occurs in the head, neck, and axilla. Intraabdominal lymphangioma is very rare and comprises less than 5% of all lymphangiomas. From 50% to 60% of cystic lymphangioma cases present symptoms by age 1 year and 90% of the cases present by age 2 years [3]. The aetiology of lymphangioma is poorly understood but is generally believed to be a developmental abnormality associated with failure of the developing lymphatic tissues to establish normal communication with regional lymphatic drainage, resulting in dilatation of the abnormal channels [11]. Clinical presentation [12] may be chronic in which there is gradual distention in abdomen with or without pain. It may present in acute form in which there is acute pain, distention, fever, vomiting and peritonitis. There may be features of small bowel obstruction which may occur by extrinsic luminal compression, by traction on the mesentery or by volvulus.

Plain radiographs may show a non-calcified soft-tissue mass, displacement of intestinal loops, or features of small bowel obstruction [13]. A well-circumscribed anechoic mass with posterior acoustic enhancement is a typical ultrasound presentation [14]. Computed tomography scan shows well defined, thin walled multiseptate lesion and distinguished from ascites by the absence of bowel loop separation or fluid in the typical sites, such as the cul-de-sac [15]. The cyst wall and septa can show enhancement after intravenous injection of contrast agent. Calcification is uncommon [11]. On magnetic resonance imaging, lymphangiomas have a low signal intensity on T1-weighted images and a high signal intensity on T2-weighted images. If haemorrhage or infection occurs, the CT attenuation and magnetic resonance imaging signal pattern may alter [11]. Complications are intestinal obstruction (most common), volvulus, haemorrhage into the cyst, infection, rupture, cystic torsion and obstruction of the urinary and biliary tract [2].

Abdominal lymphangiomas should be differentiated from other abdominal cystic masses such as cystic teratomas, mucinous cystadenomas, bronchogenic cysts, ovarian cysts, nonpancreatic pseudocysts, and complicated ascites [3, 16]. The presence of septa, compression on adjacent intestinal loops and lack of fluid in a dependent recess of the peritoneum and between leaves of the small-bowel mesentery suggests lymphangioma [15]. Complete resection with negative microscopic margins is the treatment of choice, even when asymptomatic, and any involved organ must also be resected [17]. Radical resection is sometimes technically impossible, because of local invasiveness with infiltration of adjacent organs or the main arterial branches. Incomplete resection has a 10% postoperative recurrence rate. Simple aspiration with or without injection of a sclerosing agent should be avoided because of extremely high risk of infection or recurrence rates [18]. If cysts are discovered prenatally, intervention during early infancy is indicated to prevent potential complications such as obstruction and intestinal volvulus. Laparoscopic resection is an excellent alternative to conventional open abdominal resection, and gives the patients the advantages of minimally invasive surgery [19]. In our patient the cyst was found in the greater omentum which was excised completely without omentectomy or bowel resection. Follow-up imaging is advised, with ultrasound as the modality of choice.

## Conclusion

In a case of large cystic mass with thin walls, internal echogenicities and septations, the diagnosis of omental lymphangioma should be suspected, but histological diagnosis is necessary for definitive diagnosis. This rare case is presented because of its unusual presentation with huge size and acute intracystic haemorrhage.

## References

[1] Chateil JF, Brun M, Vergnes P, Andrieu de Lewis, Pérel Y, Diard F (2002). Abdominal cystic lymphangiomas in children: presurgical evaluation with imaging. Eur J Pediatr Surg.

[2] Hancock BJ, St-Vil D, Luks FI, Di Lorenzo M, Blanchard H (1992). Complications of lymphangiomas in children. J Pediatr Surg.

[3] Wilson SR, Bohrer S, Losada R, Price AP (2006). Retroperitoneal lymphangioma: an unusual location and presentation. J Pediatr Surg.

[4] Hebra A, Brown MF, McGeehin KM, Ross AJ (1993). Mesenteric, omental, and retroperitoneal cysts in children: a clinical study of 22 cases. South Med J.

[5] Okur H, Kucukaydin M, Ozokutan BH, Durak AC, Kazez A, Kose O (1997). Mesenteric, omental, and retroperitoneal cysts in children. Eur J Surg.

[6] Pang LC (1990). Acute abdominal conditions in mesenteric lymphangioma. South Med J.

[7] Rifenburg NE, Batton B, Vade A (2006). Ruptured retroperitoneal lymphatic malformation. Comput Med Imaging Graph.

[8] Porras-Ramirez G, Hernandez-Herrera MH (1991). Hemorrhage into mesenteric cyst following trauma as a cause of acute abdomen. J Pediatr Surg.

[9] Rieker RJ, Quentmeier A, Weiss C, Kretzschmar U, Amann K, Mechtersheimer G (2000). Cystic lymphangioma of the smallbowel mesentery. Pathol Oncol Res.

[10] Neumann DP, Henken EM (1997). Lymphangioma of the jejunal mesentery. J Ultrasound Med.

[11] Ko SF, Ng SH, Shieh CS, Lin JW, Huang CC, Lee TY (1995). Mesenteric cystic lymphangioma with myxoid degeneration: unusual CT and MR manifestations. Pediatr Radiol.

[12] De Perrot M, Rostan O, Morel P, Le Coultre C (1998). Abdominal lymphangioma in adults and children. Br J Surg.

[13] Ros PR, Olmsted WW, Moser RP, Dachman AH, Hjermstad BH, Sobin LH (1987). Mesenteric and omental cysts histologic: classification with imaging correlation. Radiology.

[14] Chou YH, Tiu CM, Lui WY, Chang T (1991). Mesenteric and omental cysts: An ultrasonographic and clinical study of 15 patients. Gastrointest Radiol.

[15] Lugo-Oliveri CH, Taylor GA (1993). CT differentiation of large abdominal lymphangiomas from ascites. Pediatr Radiol.

[16] Yang DM, Jung DH, Kim H, Kang JH, Kim SH, Hwang HY (2004). Retroperitoneal cystic masses: CT, clinical, and pathologic findings and literature review. Radiographics.

[17] Tezuka K, Ogawa Y, Satake K, Ohira M, Yamada S, Uno H (2002). Lymphangioma of the lesser omentum associated with abdominal esophageal carcinoma: report of a case. Surg Today.

[18] Steayert H, Guitard J, Moscovici J, Juricic M, Vaysse P, Juskiewenski S (1996). Abdominal cystic lymphangioma in children: benign lesions that can have a proliferative course. J Pediatr Surg.

[19] Yoichi Sakurai, Keizo Taniguchi, Ichiro Uyama, Inaba Kazuki (2019). Laparoscopic exicision of the cystic Lymphangioma occurred in the lesser omentum. Surg Laparosc Endosc Percutan Tech.

